# Predictors of smoking abstinence among diabetic smokers: Evidence from the French national smoking cessation registry CDTnet

**DOI:** 10.1371/journal.pone.0321764

**Published:** 2025-06-20

**Authors:** Ingrid Allagbé, David Baudoin, Bastien Rance, Anne-Laurence Le Faou

**Affiliations:** 1 Outpatient Addictology Center, Georges Pompidou European Hospital, AP-HP, Université Paris Cité, Paris, France; 2 Groupement d’Intérêt Scientifique du Réseau Français d’Excellence de Recherche sur Tabac, nicotine et produits connexes (GIS REFERTab), Paris, France; 3 Department of Medical Informatics, Georges Pompidou European Hospital, AP-HP, Université Paris Cité, Paris, France; 4 INSERM, UMRS, Centre de Recherche des Cordeliers, Université Paris Cité, Paris, France; 5 Fédération Hospitalo-Universitaire Network of Research in Substance Use Disorders, Ile-de-France, France; Gaziosmanpaşa Training and Research Hospital: Gaziosmanpasa Egitim ve Arastirma Hastanesi, TÜRKIYE

## Abstract

Evidence suggests that diabetic smokers are less likely to quit smoking when compared to non-diabetic smokers. This study aimed to determine predictors of abstinence among diabetic smokers attending smoking cessation services (SCSs) across France. We analysed data from 94,827 adult smokers registered in the French national smoking cessation registry (CDTnet) between 2007 and 2016. Participants attended ≥2 SCS visits, and one-month continued abstinence was confirmed by carbon monoxide measurement. Among 6,405 diabetic smokers, 33% achieved one-month continued abstinence, versus 38% among 88,422 non-diabetic smokers (p < 0.001). Positive predictors of abstinence among diabetic smokers included employment (odds ratio [OR], 1.61; 95% confidence interval [CI], 1.37–1.89), ≥ 3 prior quit attempts (OR, 1.61; 95% CI, 1.36–1.91), low nicotine dependence (OR, 1.42; 95% CI, 1.13–1.78), prescription of combined nicotine replacement therapy (NRT) (OR, 1.43; 95% CI, 1.19–1.72) or varenicline (OR, 1.64; 95% CI, 1.20–2.25), and ≥7 SCS consultations (OR, 4.47; 95% CI, 3.40–5.84). Conversely, negative predictors included history of myocardial infarction/angina (OR, 0.82; 95% CI, 0.70–0.96), chronic bronchitis/chronic obstructive pulmonary disease (OR, 0.79; 95% CI, 0.69–0.91), anxiety (OR, 0.82; 95% CI, 0.71–0.94), recent cannabis use (OR, 0.67; 95% CI, 0.50–0.90), and exclusive oral NRT use (OR, 0.70; 95% CI, 0.56–0.88). Overall, tailored cessation programs are crucial for enhancing cessation outcomes among diabetic smokers.

## Introduction

Smoking is a major risk factor for diabetes, with a well-established dose-dependent effect [1,2]. Additionally, smoking impairs insulin sensitivity, which in turn affects glycemic control and contributes to the development and progression of diabetes [3–5]. Smokers with diabetes face an elevated risk of cardiovascular events and all-cause mortality compared to non-smokers with diabetes [6,7]. The increased risk extends to microvascular and macrovascular complications [7,8].

In the management of diabetic smokers, smoking cessation represents a priority to mitigate long-term health risks [3,9]. Despite international recommendations emphasising the benefits of quitting smoking for individuals with diabetes [1,10], smoking cessation rates remain low compared to those without diabetes [3,11,12]. Moreover, there has been limited effort in developing and evaluating tailored interventions specifically designed to support smoking cessation in individuals with diabetes [3,9]. Identifying predictors of smoking abstinence enables the tailoring of smoking cessation programs to meet the specific needs of people with diabetes [3]. By analysing data from the French national smoking cessation registry CDTnet, this study aims to determine the factors associated with smoking abstinence in diabetic smokers, with the ultimate goal of developing tailored interventions to enhance their cessation rates.

## Methods

### Study design

A retrospective study was performed using real-life data collected between 2007 and 2016 from smokers registered in the French national smoking cessation registry (*Consultation de Dépendance Tabagique* [CDTnet]; www.cdtnet.fr). CDTnet collects anonymised information on smokers who visit a smoking cessation service (SCS) located across France. SCSs in France are often situated within hospital settings. SCSs offer behavioural support and pharmacological treatment, with the first visit lasting 45–60 minutes and follow-up visits around 30 minutes. The support includes various behavioural change techniques, as per the Michie et al. taxonomy [13], such as action planning, goal setting, reviewing outcome goals, pharmacological support if accepted, information about health consequences of smoking and smoking cessation, and biofeedback (carbon monoxide [CO] monitoring) [14]. Pharmacological support aligns with recommendations for the general population, with nicotine replacement therapy (NRT) recommended as a first-line treatment by the French National Authority for Health. Additionally, varenicline and bupropion are the only drugs with marketing authorization for smoking cessation in France [15].

The authors obtained permission from the French independent administrative authority responsible for protecting privacy and personal data, the *Commission Nationale de l’Informatique et des Libertés* (CNIL: National Commission for Information Technology and Civil Liberties) (authorisation number 739406), to collect information from CDTnet. As the study was retrospective, and all data were collected from individuals receiving routine treatment and anonymised, specific ethical approval was not required. Smokers provided written informed consent before their registration on CDTnet.

### Study population

**Fig 1:**
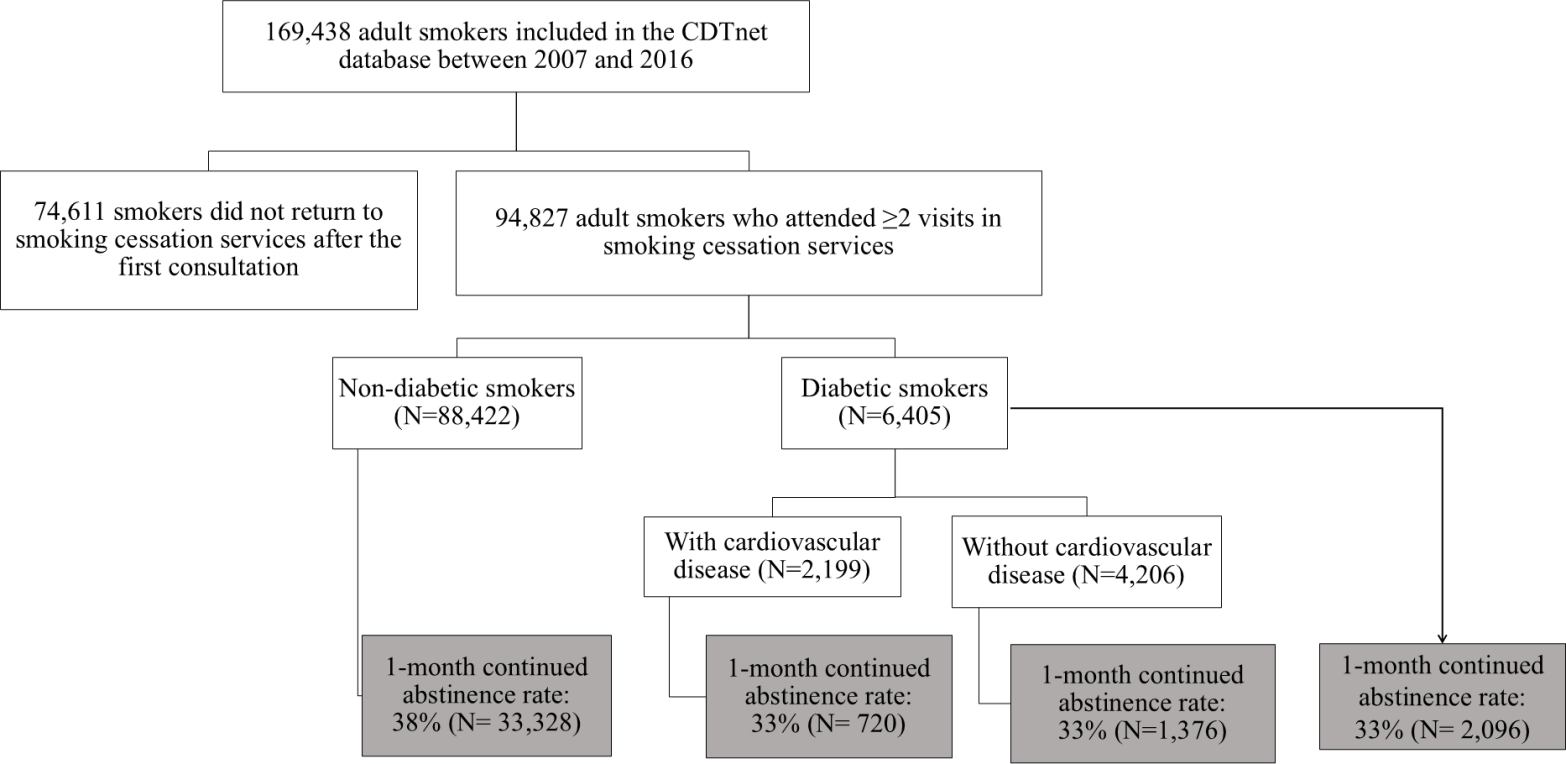
Flowchart of the study population.

This study included current smokers, defined as adults (≥18 years) who reported daily or intermittent combustible tobacco use (cigarillo, rolled cigarette, manufactured cigarette) at their initial visit to a SCS, and who attended ≥2 SCS visits [16]. We included participants with at least two SCS visits because about 50% of smokers who visit a SCS do not return for follow-up [17]. The inclusion period was from January 1, 2007 to December 31, 2016. Pregnant women were excluded. During our study period, 264 SCSs were available across 79 departments in France, comprising 93.2% public hospitals, 2% private hospitals, 1.6% prison health services, 2.4% addiction centers, 0.5% general practitioner practices, and 0.3% medical dispensaries. Of 169,438 adult smokers registered in the CDTnet database between 2007 and 2016, 94,827 (56.0%) attended ≥2 SCS visits, and were hence included in the present analysis (Fig 1).

### Data

Collected data at baseline included sociodemographic information (sex, age, educational level, employment status), medical history, and smoking behaviours. All collected information, including medical history, was self-reported by the smokers and checked by the SCS staff, before being registered in CDTnet.

Age was analysed both as a continuous variable and a categorical variable, in four groups (18–29, 30–49, 50–69, ≥ 70 years). Employment status was defined by four classes (employed, retired, unemployed, training/student). The educational level was also defined by four categories (no diploma, vocational school diploma, high school diploma, and higher education). Medical history included cardiovascular risk factors (diabetes, body mass index [BMI] ≥25 kg/m^2^ for overweight/obesity, arterial hypertension, and/or hypercholesterolemia), cardiovascular diseases (CVDs) (angina pectoris or myocardial infarction [MI], stroke, and/or peripheral arterial disease [PAD]), respiratory diseases (chronic bronchitis, chronic obstructive pulmonary disease [COPD], and/or asthma), smoking-related cancers (lung, otorhinolaryngology, bladder), and psychiatric disorders (depression history, depression symptoms, anxiety symptoms). The Hospital Anxiety and Depression (HAD) scale was used to screen for anxiety or depressive disorders. A HAD anxiety score ≥11 suggests anxiety, and a HAD depression score ≥8 suggests depression [18].

Smoking behaviour variables included reasons for visiting a SCS (self-referral, contact with the hospital whether during hospitalisation or during a specialised consultation, referral by a primary care health professional, or request from family and friends), indoor smoking, presence of other smokers at home, number of previous quit attempts lasting ≥7 days, and number of cigarettes smoked per day. To determine the number of cigarettes smoked per day, we used the tobacco equivalences published by the French Public Health Agency: one cigarillo = two rolled cigarettes = two manufactured cigarettes [19]. We also collected information on nicotine dependence, assessed by the heaviness of the smoking index (HSI) [**20**]. The HSI is a self-report measure derived from the Fagerström test for nicotine dependence, including two questions: number of cigarettes smoked daily and time from waking up to the first cigarette of the day. A HSI score ≥4 indicates high nicotine dependence [20]. Self-confidence in quitting was further measured by a visual analogue scale from 0 to 10. Self-confidence was classified as low for scores of 0‒4, medium for scores of 5‒6, and high for a score ≥7. In addition, we recorded E-cigarette use, opioid substitution treatment, as well as alcohol misuse and cannabis consumption in the last 30 days. Smoking cessation treatments (cognitive behavioural techniques, NRT, varenicline, bupropion) prescribed during the initial visit to the SCS were also registered in CDTnet.

### Smoking status assessment

During follow-up visits at the SCS, smoking cessation specialists measured CO levels in exhaled air. The quit date was defined as the date when the smoker returned for a follow-up consultation and declared being a non-smoker, with smoking abstinence validated by a CO measure. While the smoker may have quit before the follow-up consultation, this timepoint was chosen to prevent misclassification. Independently from the follow-up duration, we adopted a criterion of one-month continuous abstinence, which reflects that the individual had not smoked at all for 28 days after the quit date. This information was assessed by the SCS staff during follow-up consultations. In addition, smoking abstinence was confirmed by an expired CO measurement <5 parts per million (ppm) at each follow-up visit, according to the criteria used in the literature [21,22]. This threshold was also based on a boxplot that we produced to determine the median CO levels of smokers who had declared they had quit and those who had not. Median CO was 14 ppm for non-quitters and 3 ppm for quitters (S1 Fig). We also adopted the criteria used in real-life settings for evaluating the effectiveness of English SCSs, showing that one-month continued abstinence allows to predict one-year abstinence [22,23]. Smokers who reported reducing their tobacco consumption during the follow-up period by ≥50% relative to levels at the first consultation were considered to have a reduced smoking status [24]. Smokers lost to follow-up, unless they succeeded to quit, were considered smokers [25].

### Statistical analyses

Descriptive statistics were used to compare baseline information and one-month continued abstinence between diabetic smokers and non-diabetic smokers, and between diabetic smokers with CVDs (i.e., MI/angina, stroke, or PAD) and diabetic smokers without CVDs. A Student’s t-test or a Mann-Whitney U test was used for continuous variables, and chi-square test was used for categorical variables. The results of the analyses were described by mean and standard deviation (SD) for continuous variables and by numbers and percentages for categorical variables. Predictors of abstinence were assessed for the two groups, diabetic smokers and non-diabetic smokers, using unconditional univariate logistic regression, multivariate logistic regression, and stepwise multivariate logistic regression. Multicollinearity was tested using the variance inflation factor. In addition, we performed a subgroup analysis to investigate factors associated with smoking abstinence in diabetic smokers with CVDs and in those without CVDs. We chose to separate diabetic smokers with CVDs from those without CVDs due to the well-established increased risk of cardiovascular complications in people with diabetes [26]. Furthermore, given that smoking is a major risk factor for CVDs, this subgroup analysis provides insights into whether the presence of CVDs in diabetic smokers impacts smoking cessation outcomes [27]. In all four regression models, the comparison group was the non-abstainer group (abstainers versus non-abstainers).

Two-tailed p-values <0.05 and confidence intervals (CI) of odds ratios (OR) not inclusive of 1 were considered statistically significant. All statistical analyses were performed using R (version 4.0.3; R Foundation for Statistical Computing, Vienna, Austria).

## Results

### Characteristics of participants at baseline

Our study included 94,827 adult smokers who attended a mean±SD of 4 ± 5 visits to a SCS between 2007 and 2016. Of these participants, 6,405 (7%) were diabetic smokers, and 88,422 (93%) were non-diabetic smokers. Among the 6,405 diabetic smokers, 2,199 (34%) had a previous or a current CVD (MI/angina, stroke, and/or PAD), while 4,206 (66%) did not (Fig 1). The mean follow-up duration was 129.7 days, with a median of 37 days.

Table 1 presents the sociodemographic and clinical characteristics of the overall study population and diabetic smokers compared to non-diabetic smokers. Compared to non-diabetic smokers, diabetic smokers were older (mean±SD age, 54 ± 11 versus 46 ± 12 years; p < 0.001), less likely to be women (36% versus 50%; p < 0.001), had a lower prevalence of higher education (19% versus 31%; p < 0.001), and were more likely to be unemployed (26% versus 18%; p < 0.001). Clinically, diabetic smokers exhibited a higher prevalence of cardiovascular risk factors, chronic bronchitis/COPD, and psychiatric disorders compared to non-diabetic smokers. The prevalence of CVDs was also significantly higher in diabetic smokers than non-diabetic smokers, with rates of 20% versus 6% for MI/angina, 8% versus 3% for stroke, and 15% versus 5% for PAD, respectively (all p < 0.001).

**Table 1:** Sociodemographic and clinical characteristics of the study population of 94,827 smokers who attended ≥2 visits in smoking cessation services.

Characteristics	Overall (N = 94,827)	Diabetic persons (N = 6,405)	Non-diabetic persons (N = 88,422)	p-value
**Sex, n (%)**				**<0.001**
Women	46,947 (50)	2,330 (36)	44,617 (50)	
Men	47,880 (50)	4,075 (64)	43,805 (50)	
**Age in years, mean ± standard deviation**	47 ± 12	54 ± 11	46 ± 12	**<0.001**
**Age in years, n (%)**				**<0.001**
18–29	9,362 (10)	169 (3)	9,193 (10)	
30–49	45,260 (48)	1,866 (29)	43,394 (49)	
50–69	37,999 (40)	4,046 (63)	33,953 (38)	
≥70	2,206 (2)	324 (5)	1,882 (3)	
**Education, n (%)**				**<0.001**
No diploma	20,642 (22)	2,107 (33)	18,535 (21)	
Vocational school diploma	25,966 (27)	2,027 (32)	23,939 (27)	
High school diploma	19,483 (21)	1,068 (17)	18,415 (21)	
Higher education	28,736 (30)	1,203 (19)	27,533 (31)	
**Employment status, n (%)**				**<0.001**
Employed	56,794 (60)	2,494 (39)	54,300 (61)	
Retired	12,403 (13)	1,695 (26)	10,708 (12)	
Unemployed	17,225 (18)	1,688 (26)	15,537 (18)	
In training/student	8,405 (9)	528 (9)	7,877 (9)	
**Cardiovascular risk factors, n (%)**				
Body mass index ≥25 kg/m^2^	36,367 (38)	4,228 (66)	32,136 (36)	**<0.001**
Arterial hypertension	14,071 (15)	3,023 (47)	11,048 (12)	**<0.001**
Hypercholesterolemia	17,331 (18)	2,878 (45)	14,453 (16)	**<0.001**
**Cardiovascular diseases, n (%)**				
Myocardial infarction/angina	6,994 (7)	1,267 (20)	5,727 (6)	**<0.001**
Stroke	3,393 (4)	483 (8)	2,910 (3)	**<0.001**
Peripheral arterial disease	5,248 (6)	941 (15)	4,307 (5)	**<0.001**
**Respiratory diseases, n (%)**				
Chronic bronchitis/chronic obstructive pulmonary disease	18,829 (20)	1,891 (30)	16,938 (19)	**<0.001**
Asthma	11,281 (12)	826 (13)	10,455 (12)	**0.010**
**Cancers**	4,078 (4)	293 (5)	3,785 (4)	0.30
**Psychiatric disorders, n (%)**				**<0.001**
Depression history	26,656 (28)	1,968 (31)	24,688 (28)	
Anxiety symptoms	26,220 (28)	2,122 (33)	24,098 (27)	**<0.001**
Depression symptoms	33,066 (35)	2,024 (32)	31,042 (35)	**<0.001**

Diabetic smokers and non-diabetic smokers demonstrated distinct motivations for seeking smoking cessation consultation (Table 2). Non-diabetic smokers were more often self-referred to a SCS (46% versus 26% for diabetic smokers; p < 0.001). Conversely, diabetic smokers were more frequently referred to a SCS after hospital contact (55% versus 34% for non-diabetic smokers; p < 0.001). Diabetic smokers also displayed heavier smoking patterns, with a mean±SD number of cigarettes smoked per day of 28 ± 19 in diabetic smokers versus 24 ± 16 for non-diabetic smokers (p < 0.001). In addition, 49% of diabetic smokers reported smoking >20 cigarettes per day versus 39% of non-diabetic smokers (p < 0.001). Furthermore, diabetic smokers scored higher on the HSI, with 62% showing high nicotine dependence compared to 58% in non-diabetic smokers (p < 0.001). Among diabetic smokers, 33% achieved a one-month continued abstinence from smoking, whereas this rate was 38% in non-diabetic smokers (p < 0.001). Additionally, diabetic smokers with and without CVDs both attained the same one-month continued abstinence rate of 33% (Fig 1). Concerning smoking cessation treatments, combined NRT (nicotine patch with an oral form of NRT) emerged as the most commonly prescribed cessation treatment not only in the overall study population (45%) but also in the two groups of smokers, diabetic and non-diabetic smokers (Table 2).

**Table 2:** Smoking characteristics in the study population of 94,827 smokers who attended ≥2 visits in smoking cessation services.

Characteristics	Overall (N = 94,827)	Diabetic persons (N = 6,405)	Non-diabetic persons (N = 88,422)	p-value
**Reason for smoking consultation, n (%)**				**<0.001**
Self-referral	42,608 (45)	1,696 (26)	40,912 (46)	
Hospital contact	33,439 (35)	3,529 (55)	29,910 (34)	
Referred by a primary care health professional	14,011 (15)	972 (15)	13,039 (15)	
Encouraged by entourage	4,769 (5)	208 (4)	4,561 (5)	
**Smokes at home, n (%)**	3,167 (3)	287 (4)	2,880 (3)	**<0.001**
**Other smokers at home, n (%)**	2,072 (2)	156 (2)	1,916 (2)	0.20
**Number of prior attempts to quit, n (%)**				**<0.001**
0	28,826 (30)	2,018 (32)	26,808 (30)	
1 − 2	44,708 (47)	3,095 (48)	41,613 (47)	
≥3	21,293 (23)	1,292 (20)	20,001 (23)	
**Number of cigarettes smoked per day, n (%)**				**<0.001**
Mean ± standard deviation	25 ± 16	28 ± 19	24 ± 16	
≤10	15,412 (16)	961 (15)	14,451 (16)	
11–20	41,494 (44)	2,331 (36)	39,163 (44)	
21–40	29,320 (31)	2,300 (36)	27,020 (31)	
≥41	8,601 (9)	813 (13)	7,788 (9)	
**Heaviness of smoking index (nicotine dependence), n (%)**				**<0.001**
Low: 0 − 1	10,318 (11)	595 (10)	9,723 (11)	
Moderate: 2 − 3	29,258 (31)	1,807 (28)	27,451 (31)	
High: 4 − 6	55,251 (58)	4,003 (62)	51,248 (58)	
**Confidence in ability to quit, n (%)**				0.60
Low: 0 − 4	30,194 (32)	2,008 (31)	28,186 (32)	
Moderate: 5 − 6	32,466 (34)	2,220 (35)	30,246 (34)	
High: 7 − 10	32,167 (34)	2,177 (34)	29,990 (34)	
**Cannabis consumption in the last 30 days, n (%)**	10,239 (11)	351 (5)	9,888 (11)	**<0.001**
**≥2 glasses of alcohol per day, n (%)**	13,659 (14)	1,005 (16)	12,654 (14)	**0.002**
**E-cigarette user, i.e., dual user, n (%)**	1,911 (2)	148 (2)	1,763 (2)	0.081
**Opioid substitution treatment, n (%)**	1,283 (1)	74 (1)	1,209 (1)	0.20
**Smoking cessation medications, n (%)**				**<0.001**
No pharmacotherapy (cognitive behavioural techniques)	14,298 (15)	858 (13)	13,440 (15)	
Transdermal nicotine patches	16,265 (17)	1,173 (18)	15,092 (17)	
Oral nicotine substitute	13,849 (15)	1,049 (16)	12,800 (14)	
Combined nicotine replacement therapy	42,950 (45)	2,975 (46)	39,975 (45)	
Varenicline	6,158 (6)	277 (4)	5,881 (7)	
Varenicline with nicotine replacement therapy	1,094 (1)	66 (1)	1,028 (1)	
Bupropion with or without nicotine replacement therapy	213 (0.2)	7 (0.1)	206 (0.2)	
**Number of follow-up smoking consultations, n (%)**				
Mean ± standard deviation	4 ± 5	4 ± 5	4 ± 5	**0.001**
1–3	62,305 (66)	4,275 (67)	58,030 (66)	**0.021**
4–6	18,748 (20)	1,181 (18)	17,567 (20)	
≥7	13,774 (15)	949 (15)	12,825 (15)	
**Smoking status at one-month follow-up by carbon monoxide, n (%)**				**<0.001**
Continued abstinence	35,424 (37)	2,096 (33)	33,328 (38)	
Smoking reduction	31,159 (33)	2,301 (36)	28,858 (33)	
Non-abstinence	28,244 (30)	2,008 (31)	26,236 (30)	

### Predictors of abstinence

Our multivariate analysis identified several common factors associated with smoking abstinence in both diabetic smokers and non-diabetic smokers (Table 3). Notably, age and sex did not impact one-month continued abstinence in both groups. Positive factors for smoking abstinence among diabetic smokers included: being employed (OR, 1.61; 95% CI, 1.37–1.89; p < 0.001) or retired (OR, 1.54; 95% CI, 1.25–1.89; p < 0.001), self-referral to SCSs (OR, 1.19; 95% CI, 1.03–1.37; p = 0.018), having prior quit attempts (OR, 1.61 for ≥3 prior attempts; 95% CI, 1.36–1.91; p < 0.001), presenting with low nicotine dependence (OR, 1.42; 95% CI, 1.13–1.78; p = 0.003) and high confidence in quitting (OR, 1.21; 95% CI, 1.05–1.40; p = 0.011), being prescribed transdermal nicotine patches (OR, 1.26; 95% CI, 1.02–1.56; p = 0.033), combined NRT (OR, 1.43; 95% CI, 1.19–1.72; p < 0.001), or varenicline (OR, 1.64; 95% CI, 1.20–2.25; p = 0.002) as smoking cessation treatments, and having ≥4 follow-up consultations (OR, 4.47 for ≥7 follow-up consultations; 95% CI, 3.40–5.84; p < 0.001). By contrast, negative factors for smoking abstinence in diabetic smokers were: having MI/angina (OR, 0.82; 95% CI, 0.70–0.96; p = 0.012), chronic bronchitis/COPD (OR, 0.79; 95% CI, 0.69–0.91; p = 0.001), depression history (OR, 0.84; 95% CI, 0.73–0.98; p = 0.025), anxiety symptoms (OR, 0.82; 95% CI, 0.71–0.94; p = 0.005), cannabis use in the last 30 days (OR, 0.67; 95% CI, 0.50–0.90; p = 0.008), and being prescribed oral forms of NRT as the only smoking cessation medication (OR, 0.70; 95% CI, 0.56–0.88; p = 0.002). The only factor hampering abstinence that differed between diabetic and non-diabetic smokers was the presence of MI/angina, with diabetic smokers with MI/angina having reduced chances to quit (OR, 0.82; 95% CI, 0.70–0.96; p = 0.012) compared to non-diabetic smokers (OR, 1.02; 95% CI, 0.96–1.10; p = 0.50).

**Table 3:** Multivariate analysis of smoking abstinence in diabetic persons (N = 6,405) and in non-diabetic persons (N = 88,422).

	Diabetic persons (N = 6,405)			Non-diabetic persons (N = 88,422)		
Characteristics	Odds ratio	95% CI	p-value	Odds ratio	95% CI	p-value
Women	—	—		—	—	
Men	1.13	0.98–1.29	0.084	0.99	0.96–1.02	0.50
**Age**						
18–29	—	—		—	—	
30–49	1.24	0.76–2.04	0.40	1.02	0.95–1.09	0.70
50–69	1.31	0.73–2.41	0.40	0.96	0.86–1.08	0.50
≥70	1.46	0.68–3.15	0.30	1.04	0.87–1.25	0.70
**Education**						
No diploma	—	—		—	—	
Vocational school diploma	1.08	0.93–1.26	0.30	**1.10**	**1.05–1.16**	**<0.001**
High school diploma	1.01	0.84–1.21	>0.90	**1.14**	**1.08–1.20**	**<0.001**
Higher education	0.90	0.75–1.08	0.30	**1.15**	**1.10–1.21**	**<0.001**
**Employment status**						
Unemployed	—	—		—	—	
Employed	**1.61**	**1.37–1.89**	**<0.001**	**1.64**	**1.56–1.72**	**<0.001**
Retired	**1.54**	**1.25–1.89**	**<0.001**	**1.66**	**1.55–1.78**	**<0.001**
In training/student	**1.32**	**1.03–1.69**	**0.029**	**1.16**	**1.08–1.24**	**<0.001**
**Reason for smoking consultation**						
Hospital contact	—	—		—	—	
Self-referral	**1.19**	**1.03–1.37**	**0.018**	**1.10**	**1.06–1.14**	**<0.001**
Referred by a primary care health professional	1.18	1.00–1.40	0.055	0.99	0.94–1.04	0.60
Encouraged by entourage	1.01	0.72–1.40	>0.90	0.96	0.89–1.04	0.30
**Smokes at home**	0.85	0.61–1.19	0.30	**0.80**	**0.72–0.89**	**<0.001**
**Other smokers at home**	0.84	0.53–1.30	0.40	**0.80**	**0.70–0.90**	**<0.001**
**Cardiovascular risk factors**						
Body mass index ≥25 kg/m^2^	1.11	0.76–1.64	0.60	**1.12**	**1.04–1.20**	**0.002**
Arterial hypertension	1.07	0.95–1.21	0.30	0.99	0.94–1.04	0.70
Hypercholesterolemia	1.09	0.96–1.23	0.20	1.04	1.00–1.09	0.052
**Cardiovascular diseases**						
Myocardial infarction/angina	**0.82**	**0.70–0.96**	**0.012**	1.02	0.96–1.10	0.50
Stroke	1.18	0.95–1.47	0.14	1.01	0.92–1.10	0.80
Peripheral arterial disease	0.93	0.78–1.10	0.40	**0.92**	**0.85–0.99**	**0.026**
**Respiratory diseases**						
Chronic bronchitis/chronic obstructive pulmonary disease	**0.79**	**0.69–0.91**	**0.001**	**0.87**	**0.83–0.90**	**<0.001**
Asthma	0.99	0.82–1.20	>0.90	**0.94**	**0.89–0.99**	**0.017**
**Cancers**	0.89	0.66–1.18	0.40	**0.91**	**0.84–0.98**	**0.018**
**Psychiatric disorders**						
Depression history	**0.84**	**0.73–0.98**	**0.025**	**0.89**	**0.86–0.93**	**<0.001**
Anxiety symptoms	**0.82**	**0.71–0.94**	**0.005**	**0.80**	**0.77–0.83**	**<0.001**
Depression symptoms	0.92	0.80–1.06	0.30	**0.91**	**0.88–0.94**	**<0.001**
**Number of prior attempts to quit**						
0	—	—		—	—	
1–2	**1.27**	**1.10–1.46**	**<0.001**	**1.33**	**1.28–1.38**	**<0.001**
≥3	**1.61**	**1.36–1.91**	**<0.001**	**1.58**	**1.51–1.65**	**<0.001**
**Number of cigarettes smoked per day**						
≥41	—	—		—	—	
≤10	1.15	0.90–1.49	0.30	**1.21**	**1.12–1.30**	**<0.001**
11-20	1.04	0.85–1.28	0.70	**1.17**	**1.10–1.25**	**<0.001**
21–40	1.09	0.90–1.33	0.40	1.06	1.00–1.13	0.050
**Heaviness of smoking index (nicotine dependence)**						
High: 4–6	—	—		—	—	
Moderate: 2–3	**1.20**	**1.04–1.39**	**0.014**	**1.16**	**1.11–1.20**	**<0.001**
Low: 0–1	**1.42**	**1.13–1.78**	**0.003**	**1.34**	**1.26–1.42**	**<0.001**
**Confidence in ability to quit**						
Low: 0–4	—	—		—	—	
Moderate: 5–6	0.87	0.75–1.01	0.073	**0.95**	**0.91–0.99**	**0.009**
High: 7–10	**1.21**	**1.05–1.40**	**0.011**	**1.32**	**1.27–1.38**	**<0.001**
**Cannabis consumption in the last 30 days**	**0.67**	**0.50–0.90**	**0.008**	**0.71**	**0.68–0.75**	**<0.001**
**≥2 glasses of alcohol in per day**	0.85	0.72–1.01	0.064	**0.87**	**0.83–0.91**	**<0.001**
**E-cigarette user, i.e., dual user**	0.91	0.60–1.36	0.70	**1.13**	**1.01–1.26**	**0.034**
**Opioid substitution treatment**	0.54	0.26–1.03	0.076	**0.51**	**0.43–0.61**	**<0.001**
**Smoking cessation medications**						
No pharmacotherapy (cognitive behavioural techniques)	—	—		—	—	
Transdermal nicotine patches	**1.26**	**1.02–1.56**	**0.033**	**1.58**	**1.49–1.67**	**<0.001**
Oral nicotine substitute	**0.70**	**0.56–0.88**	**0.002**	**0.77**	**0.73–0.82**	**<0.001**
Combined nicotine replacement therapy	**1.43**	**1.19–1.72**	**<0.001**	**1.54**	**1.47–1.61**	**<0.001**
Varenicline	**1.64**	**1.20–2.25**	**0.002**	**2.20**	**2.05–2.36**	**<0.001**
Varenicline + nicotine replacement therapy	1.49	0.84–2.63	0.20	**1.99**	**1.72–2.29**	**<0.001**
Bupropion	1.54	0.23–11.6	0.70	**1.84**	**1.30–2.60**	**<0.001**
**Number of follow-up consultations**						
1–3	—	—		—	—	
4–6	**3.78**	**3.22–4.43**	**<0.001**	**3.35**	**3.20–3.50**	**<0.001**
≥7	**4.47**	**3.40–5.84**	**<0.001**	**3.43**	**3.15–3.74**	**<0.001**

**Fig 2:**
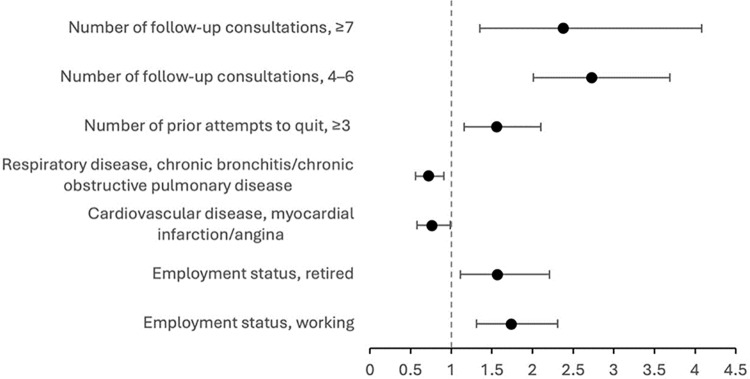
Odds ratio and 95% confidence intervals for smoking abstinence in diabetic persons with cardiovascular diseases (N = 2,199).

**Fig 3:**
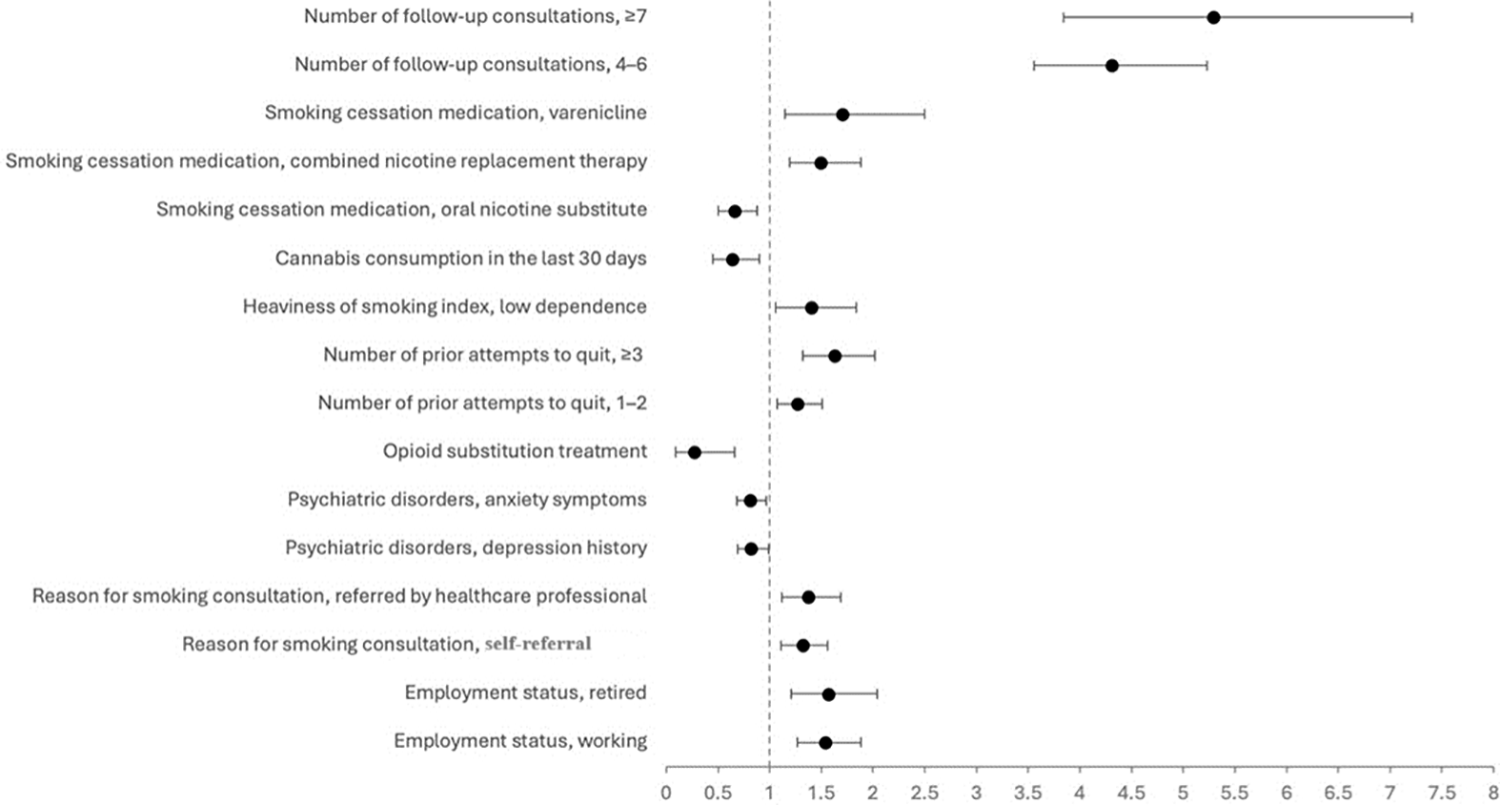
Odds ratio and 95% confidence intervals for smoking abstinence in diabetic persons without cardiovascular diseases (N = 4,206).

These positive and negative associations with smoking abstinence remained consistent in diabetic smokers with CVDs (Fig 2) and without CVDs (Fig 3). Results of the stepwise multivariate analysis of smoking abstinence among diabetic and non-diabetic smokers are presented in S1 Table.

## Discussion

In this real-world study of 94,827 French smokers seeking help to quit in a SCS, one-month continued abstinence rates were 33% among diabetic smokers and higher among non-diabetic smokers at 38% (p < 0.001). Consistently, evidence suggests that diabetic smokers are less likely to quit smoking when compared to non-diabetic smokers [11,12,28]. Moreover, diabetic smokers are less likely to be active in self-care or to comply with diabetes care recommendations [28]. This trend is reflected in the present study, in which diabetic smokers were less likely to seek help to quit on their own through SCSs compared to their non-diabetic counterparts (26% versus 46%; p < 0.001). Furthermore, diabetic smokers in this study were more frequently referred to a SCS after hospital contact than non-diabetic smokers (55% versus 34%; p < 0.001). This may be related to the higher prevalence of comorbidities among diabetic smokers, including cardiovascular risk factors, CVDs, and respiratory diseases, as well as heavier smoking patterns compared to non-diabetic smokers. Diabetic smokers were also significantly older than their non-diabetic counterparts in the current study (mean age, 54 versus 46 years; p < 0.001). This difference is in line with expectations, given that the mean age at diabetes diagnosis is 50 years [29]. Hence, smokers with diabetes might, on average, be older than those without diabetes. Of note, an earlier analysis of the CDTnet registry revealed that smokers referred to French cessation services were heavier smokers than smokers in the general population [30].

Our study highlighted several predictors influencing smoking abstinence in diabetic smokers, with or without CVDs, including employment status, prior quit attempts, low nicotine dependence, high confidence in quitting, prescription of combined NRT or varenicline as smoking cessation treatments, and multiple follow-up consultations. These results are in line with the findings of an online cross-sectional survey involving 201 smokers and ex-smokers in Australia, which highlighted the crucial role of follow-up and support in smoking cessation management as well as the effectiveness of varenicline prescription in succeeding to quit [31]. In alignment with prior research [32], we found that diabetic smokers are more likely to experience socioeconomic disadvantages, in terms of employment and education, compared to non-diabetic smokers. The impact of socioeconomic factors on smoking cessation is well-established, with individuals from lower socioeconomic backgrounds facing challenges in successfully quitting smoking, irrespective of their diabetes status [12,14,33–35]. Socioeconomic factors can influence access to resources, social support, and the ability to cope with stress, all of which can impact the success of smoking cessation efforts [12,33]. Hence, to reduce inequity in smoking cessation outcomes, multifaceted interventions are required at individual, community, and population levels, recognising the wider context of socioeconomically disadvantaged smokers and the interplay between socioeconomic status, diabetes, and smoking [12,33].

Within our study population, individuals with ≥3 prior attempts to quit smoking exhibited an approximately 60% increased likelihood of achieving smoking abstinence, regardless of diabetes status or the presence of CVDs. Smoking cessation is a chronic and dynamic process that often produces a complex back-and-forth pattern of quit attempts [36,37]. Indeed, the estimated average number of quit attempts expected before achieving long-term tobacco abstinence is 6 [38]. The most effective way to achieve smoking abstinence among diabetic smokers is to combine both behavioural and pharmacologic therapies [1,39]. Behavioural interventions are particularly important to improve self-confidence to quit [40], which has been found in our study to have a significantly positive impact on smoking abstinence. There is evidence of a robust dose-response relationship, wherein more intensive behavioural and psychological treatments, characterised by higher amounts of contact time and more sessions, yield a greater odds of sustained smoking cessation [9]. This relationship is further underscored in the present study, with the number of follow-up consultations emerging as the most impactful factor on smoking abstinence. Notably, the OR exceeded 3 for both diabetic and non-diabetic smokers, reaching 5.29 in diabetic smokers without CVDs who attended ≥7 follow-up consultations at a SCS.

The recommended first-line pharmacologic therapies for smoking cessation in both diabetic and general populations include combined NRT, varenicline, and bupropion [1,39,40]. In our study population, bupropion was rarely used (0.2%), potentially due to the fact that bupropion is not reimbursed in France [1]. Combined NRT and varenicline are considered similar in efficacy, and are regarded as the two most effective smoking cessation aids currently available [40]. Notably, combined NRT was frequently prescribed in our study, with a usage rate of 45% versus up to 17% for a single form of NRT. The rationale for combined NRT is that long-acting transdermal nicotine patches can provide a stable level of nicotine necessary to achieve and sustain cessation, while short-acting oral forms of NRT can deal with emergent nicotine cravings [1]. NRT prescription practices in French cessation services align with the literature, which has demonstrated an increased benefit on abstinence with combined NRT versus single-form NRT [41,42]. Importantly, our study discourages the use of oral nicotine substitute as the sole smoking cessation medication, as it was associated with a 30% lower likelihood of smoking abstinence among diabetic smokers.

Other negative factors for smoking abstinence among diabetic smokers in the current study included a history of MI/angina, chronic bronchitis/COPD, a history of depression, anxiety symptoms, and cannabis use within the last 30 days. In particular, the only factor that differed significantly between diabetic and non-diabetic smokers was the presence of MI/angina, highlighting the need for comprehensive smoking cessation management in diabetic smokers with MI/angina. Remarkably, earlier studies have also shown that smokers with pre-existing heart or respiratory disease were less likely to quit smoking [11,12]. One plausible explanation is that the motivation to quit smoking tends to be higher when individuals perceive a clinical trigger event as life-threatening. This motivation may be less pronounced in those who have already experienced a cardiac or respiratory event in the past [11,12]. In addition, weight gain after quitting is a common concern for individuals with diabetes and CVDs, as it may lead to poor glycemic control and increases the risk of complications [12,43,44]. Interestingly, among non-diabetic smokers in our study, having a BMI ≥ 25 kg/m^2^ was associated with a 12% higher likelihood of successful smoking cessation (95% CI, 1.04–1.20; p = 0.002). This suggests that individuals with higher BMI are less concerned about weight gain associated with quit attempts [11]. However, this association was not observed among diabetic smokers. Nonetheless, in individuals with diabetes, the cardiovascular health and mortality benefits of smoking cessation outweigh concerns about weight gain [45]. Therefore, a proactive approach is needed to manage weight gain among diabetic individuals attempting to quit smoking, especially those who smoke heavily or are prone to binge eating [45].

Smoking quit rates are also generally lower in individuals with mental health conditions, since such conditions can complicate smoking cessation efforts by affecting motivation, coping mechanisms, and overall well-being [12,28,40]. Similarly, another analysis of CDTnet revealed that recent cannabis users in France were less likely to quit smoking [14]. This underscores the potential influence of concurrent substance use, like tobacco and cannabis, on smoking behaviour and suggests that addressing multiple substance use is crucial for successful smoking cessation [46].

This study has strengths and limitations. As in any observational study, causality cannot be established, and unmeasured confounding factors could influence the observed associations. Additionally, due to the large sample size, some statistically significant results with small absolute differences should be interpreted cautiously. Moreover, our study population consisted of treatment-seeking patients attending ≥2 visits to a SCS. These results may not apply to the general diabetic population of smokers. Furthermore, although all collected information was validated by the SCS staff before being registered in CDTnet, reliance on self-reported data may introduce recall bias. Another limitation of our study is the inability to include racial or ethnic data as predictors, due to French legal restrictions that prohibit the collection of information directly revealing racial or ethnic origins. Nevertheless, our study is strengthened by the inclusion of every region in France, including overseas territories, in the CDTnet registry, which ensures a geographically diverse and nationally representative sample. In addition, smoking cessation was based on validated abstinence through CO measurement at each follow-up visit, offering a reliable assessment compared to self-reported cessation.

## Conclusions

Our study highlighted key predictors influencing smoking abstinence among individuals with diabetes across France. Positive predictors included employment, prior quit attempts, low nicotine dependence, prescription of combined NRT or varenicline, and multiple follow-up consultations. By contrast, negative predictors encompassed health conditions like MI/angina, chronic bronchitis/COPD, anxiety symptoms, recent cannabis use, and exclusive use of oral NRT. Results of the present study provide useful information for designing effective smoking cessation programs for diabetic smokers.

## Supporting information

S1 Fig Boxplot to visualize and determine the median carbon monoxide level, which was 14 parts per million (ppm) for non-quitters and 3 ppm for quitters. (TIF)

S1 Table Stepwise multivariate analysis of smoking abstinence in diabetic persons (N = 6,405) and in non-diabetic persons (N = 88,422). (DOCX)
