# Supplementary figures and images for: Predictors of smoking abstinence among diabetic smokers: Evidence from the French national smoking cessation registry CDTnet
Source: PLoS One. 2025 Jun 20;20(6):e0321764. doi: 10.1371/journal.pone.0321764 (PMC12180648; doi:10.1371/journal.pone.0321764)

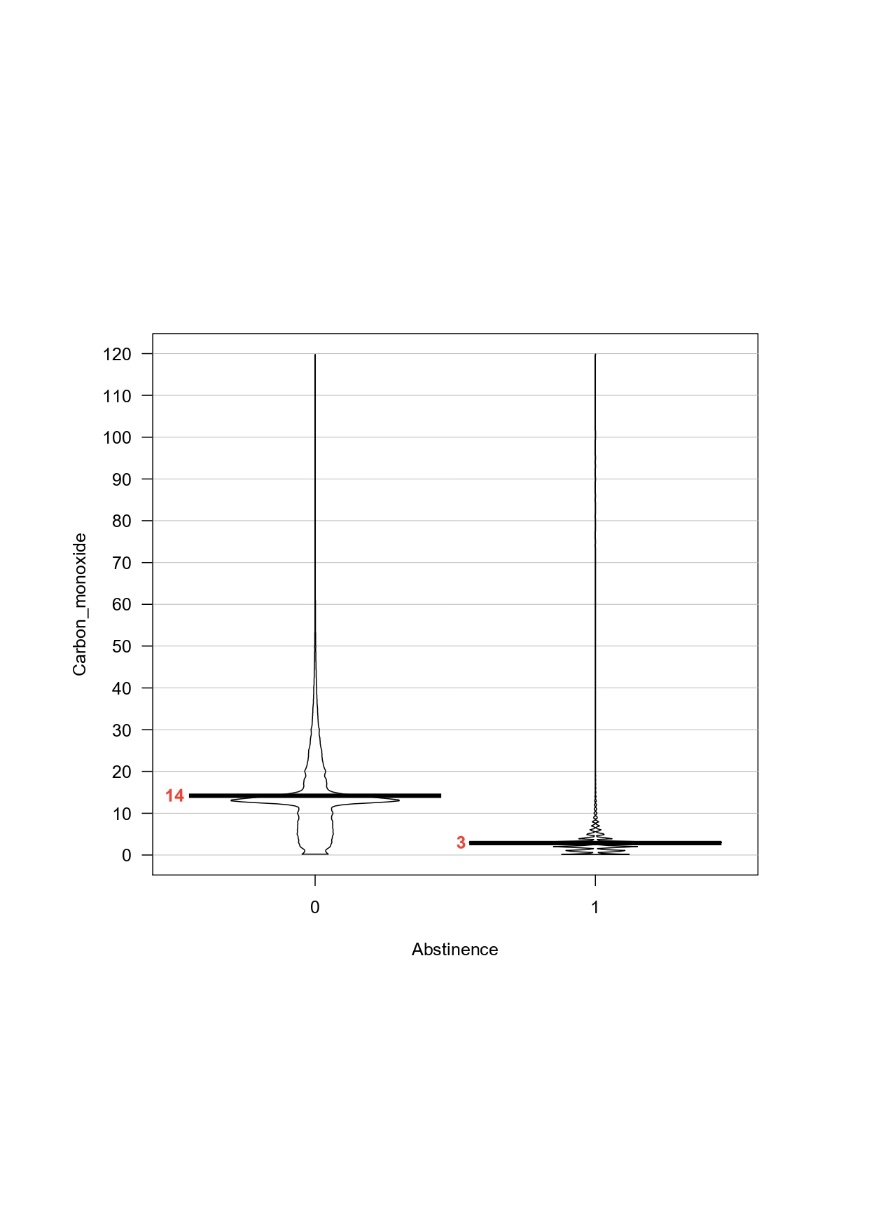

Supplement: S1 Fig — (TIF) [file pone.0321764.s001.tif]
