# Supplementary material for: Predictors of smoking abstinence among diabetic smokers: Evidence from the French national smoking cessation registry CDTnet
Source: PLoS One. 2025 Jun 20;20(6):e0321764. doi: 10.1371/journal.pone.0321764 (PMC12180648; doi:10.1371/journal.pone.0321764)
Supplement: S1 Table — (DOCX) [file pone.0321764.s002.docx]

**S1 Table. Stepwise multivariate analysis of smoking abstinence in diabetic persons (N=6,405) and in non-diabetic persons (N=88,422)**

|  | **Diabetic persons**  **(N=6,405)** | | | **Non-diabetic persons**  **(N=88,422)** | | |
| --- | --- | --- | --- | --- | --- | --- |
| **Characteristics** | **Odds ratio** | **95% CI** | **p-value** | **Odds ratio** | **95% CI** | **p-value** |
| Women | — | — |  |  |  |  |
| Men | 1.14 | 1.00–1.30 | 0.051 |  |  |  |
| **Age** | 1.01 | 1.00–1.02 | 0.043 |  |  |  |
| 18–29 |  |  |  | — | — |  |
| 30–49 |  |  |  | 1.05 | 0.99–1.11 | 0.091 |
| 50–69 |  |  |  | 1.02 | 0.96–1.09 | 0.50 |
| ≥70 |  |  |  | 1.13 | 1.00–1.29 | 0.054 |
| **Education** |  |  |  |  |  |  |
| No diploma |  |  |  | — | — |  |
| Vocational school diploma |  |  |  | 1.10 | 1.05–1.15 | <0.001 |
| High school diploma |  |  |  | 1.14 | 1.08–1.19 | <0.001 |
| Higher education |  |  |  | 1.15 | 1.10–1.21 | <0.001 |
| **Employment status** |  |  |  |  |  |  |
| Unemployed | — | — |  | — | — |  |
| Employed | 1.60 | 1.37–1.88 | <0.001 | 1.64 | 1.56–1.72 | <0.001 |
| Retired | 1.51 | 1.25–1.83 | <0.001 | 1.68 | 1.58–1.80 | <0.001 |
| In training/student | 1.31 | 1.02–1.68 | 0.031 | 1.16 | 1.08–1.24 | <0.001 |
| **Reason for smoking consultation** |  |  |  |  |  |  |
| Hospital contact | — | — |  | — | — |  |
| Self-referral | 1.18 | 1.02–1.36 | 0.024 | 1.10 | 1.06–1.14 | <0.001 |
| Referred by primary care health professional | 1.17 | 0.99–1.39 | 0.067 | 0.99 | 0.94–1.04 | 0.60 |
| Encouraged by entourage | 1.00 | 0.71–1.39 | >0.90 | 0.96 | 0.89–1.03 | 0.20 |
| **Smokes at home** | 0.77 | 0.58–1.03 | 0.082 | 0.80 | 0.72–0.89 | <0.001 |
| **Other smokers at home** |  |  |  | 0.80 | 0.70–0.90 | <0.001 |
| **Cardiovascular risk factors** |  |  |  |  |  |  |
| Body mass index ≥25 kg/m^2^ |  |  |  | 1.07 | 1.04–1.11 | <0.001 |
| Arterial hypertension |  |  |  |  |  |  |
| Hypercholesterolemia | 1.10 | 0.98–1.24 | 0.12 | 1.05 | 1.00–1.09 | 0.033 |
| **Cardiovascular diseases** |  |  |  |  |  |  |
| Myocardial infarction/angina | 0.82 | 0.70–0.96 | 0.012 |  |  |  |
| Stroke | 1.19 | 0.96–1.48 | 0.11 |  |  |  |
| Peripheral arterial disease |  |  |  | 0.92 | 0.85–0.99 | 0.026 |
| **Respiratory diseases** |  |  |  |  |  |  |
| Chronic bronchitis/chronic obstructive pulmonary disease | 0.80 | 0.70–0.91 | <0.001 | 0.87 | 0.83–0.91 | <0.001 |
| Asthma |  |  |  | 0.94 | 0.89–0.99 | 0.015 |
| **Cancers** |  |  |  | 0.91 | 0.84–0.98 | 0.020 |
| **Psychiatric disorders** |  |  |  |  |  |  |
| Depression history | 0.83 | 0.71–0.96 | 0.011 | 0.89 | 0.86–0.93 | <0.001 |
| Anxiety symptoms | 0.80 | 0.70–0.91 | <0.001 | 0.80 | 0.77– 0.83 | <0.001 |
| Depression symptoms |  |  |  | 0.91 | 0.88–0.94 | <0.001 |
| **Number of prior attempts to quit** |  |  |  |  |  |  |
| 0 | — | — |  | — | — |  |
| 1–2 | 1.27 | 1.11–1.46 | <0.001 | 1.33 | 1.28–1.38 | <0.001 |
| ≥3 | 1.60 | 1.35–1.89 | <0.001 | 1.58 | 1.51–1.66 | <0.001 |
| **Number of cigarettes smoked per day** |  |  |  |  |  |  |
| ≥41 |  |  |  | — | — |  |
| ≤10 |  |  |  | 1.21 | 1.12–1.30 | <0.001 |
| 11-20 |  |  |  | 1.17 | 1.10–1.25 | <0.001 |
| 21–40 |  |  |  | 1.07 | 1.00–1.13 | 0.047 |
| **Heaviness of smoking index (nicotine dependence)** |  |  |  |  |  |  |
| High: 4–6 | — | — |  | — | — |  |
| Moderate: 2–3 | 1.48 | 1.20–1.81 | <0.001 | 1.34 | 1.26–1.42 | <0.001 |
| Low: 0–1 | 1.21 | 1.06–1.39 | 0.005 | 1.15 | 1.11–1.20 | <0.001 |
| **Confidence in ability to quit** |  |  |  |  |  |  |
| Low: 0–4 | — | — |  | — | — |  |
| Moderate: 5–6 | 0.88 | 0.76–1.02 | 0.085 | 0.95 | 0.91–0.99 | 0.010 |
| High: 7–10 | 1.22 | 1.05–1.41 | 0.008 | 1.32 | 1.27–1.38 | <0.001 |
| **Cannabis consumption in last 30 days** | 0.66 | 0.49–0.88 | 0.006 | 0.71 | 0.67–0.75 | <0.001 |
| **≥2 glasses of alcohol in per day** | 0.85 | 0.72–1.00 | 0.049 | 0.87 | 0.83–0.91 | <0.001 |
| **E-cigarette user, i.e., dual user** |  |  |  | 1.13 | 1.01–1.27 | 0.034 |
| **Smoking cessation medications** |  |  |  |  |  |  |
| No pharmacotherapy (cognitive behavioural techniques) | — | — |  | — | — |  |
| Transdermal nicotine patches | 1.27 | 1.02–1.57 | 0.030 | 1.58 | 1.49–1.67 | <0.001 |
| Oral nicotine substitute | 0.70 | 0.56–0.88 | 0.002 | 0.77 | 0.73–0.82 | <0.001 |
| Combined nicotine replacement therapy | 1.43 | 1.19–1.73 | <0.001 | 1.54 | 1.47–1.61 | <0.001 |
| Varenicline | 1.68 | 1.22–2.30 | 0.001 | 2.20 | 2.05–2.36 | <0.001 |
| Varenicline + nicotine replacement therapy | 1.48 | 0.83–2.61 | 0.20 | 1.98 | 1.72–2.29 | <0.001 |
| Bupropion | 1.53 | 0.23–11.6 | 0.70 | 1.84 | 1.30–2.60 | <0.001 |
| Bupropion + nicotine replacement therapy | 0.00 |  | >0.90 | 3.47 | 1.87–6.51 | <0.001 |
| **Number of follow-up consultations** | 0.55 | 0.26–1.05 | 0.082 | 0.51 | 0.43–0.61 | <0.001 |
| 1–3 | — | — |  | — | — |  |
| 4–6 | 3.74 | 3.19–4.39 | <0.001 | 3.35 | 3.20–3.50 | <0.001 |
| ≥7 | 4.44 | 3.38–5.80 | <0.001 | 3.43 | 3.15–3.74 | <0.001 |
